# The Écraseur in Lithotomy

**Published:** 1861-07

**Authors:** 


THE ECRASEUR IN LITHOTOMY.—We copy the following precious bit of news from one of our London exchanges, for the benefit of our brethren in this country. We should not be surprised if we were soon to hear that this horrible instrument had been used for amputating the head. With M. Chassaignac nothing seems to be impracticable. We are persuaded that he has genius enough, if he would only deign to apply it, to pass into his patients' bellies, to diagnosticate and treat their diseases by direct inspection and contact:—

"M. Chassaignac, the inventor of the écraseur, lately performed lithotomy upon a little boy, aged three years, and used the écraseur instead of the knife. A curved trocar and canula were introduced into the prostate, either through the anal orifice or the skin above the anus, (the description does not definitely say which;) and through the canula, on the withdrawal of the trocar, the chain of the écraseur was passed, led by a fine bougie and thread. By working the screw, the chain cut, without hemorrhage, through, first, the anterior portion of the sphincter ani; secondly, that portion of the prostate which surrounds the neck of the bladder; thirdly, the whole of the membranous portion of the urethra; fourthly, the soft parts of the perineum which cover this membranous portion up to the third of an inch above the anus. This accomplished, M. Chassaignac placed the nail of his left index-finger into the groove of the staff, (which had been introduced at the commencement of the operation,) and pushed a curette into the bladder, by the aid of which instrument the forceps were introduced. A calculus, about an inch in its longest diameter, was then extracted. The child had quite recovered at the end of *six weeks*. Much might be urged against the proceedings above described, notwithstanding the success which has attended the operation; but we leave to each of our readers to calculate the amount of mischief which the écraseur may do, and to set against it the hemorrhage which generally attends lithotomy."—*London Lancet.*

